# 40-Gene Expression Profile Representative of Metastatic Risk of Squamous Cell Carcinoma in a Mohs Surgical Patient

**DOI:** 10.7759/cureus.46853

**Published:** 2023-10-11

**Authors:** Kristin N Slater, Elizabeth Ryder, Carlos Gomez-Meade

**Affiliations:** 1 Dermatology, Lincoln Memorial University DeBusk College of Osteopathic Medicine, Harrogate, USA; 2 Mohs Surgery, Oklahoma Cancer Specialists Skin Cancer Center, Broken Arrow, USA

**Keywords:** squamous cell carcinoma, cscc, cutaneous squamous cell carcinoma, nmsc, skin cancer, mohs surgery

## Abstract

This case study examines using a 40-gene expression profile (40-GEP) as an independent predictor of metastatic risk in a 74-year-old male with cutaneous squamous cell carcinoma on the scalp. The patient's previous medical history included melanoma and non-melanoma skin cancers. While conventional staging methods, such as the American Joint Committee on Cancer 8th edition (AJCC8) and Brigham and Women's Hospital (BWH) staging, indicated a higher metastatic risk, the 40-GEP testing classified the patient as low risk (Class 1 result) for metastasis within three years. The patient underwent successful Mohs surgery with no evidence of perineural invasion. This case highlights the discrepancy between current staging techniques and gene expression profile testing, demonstrating the potential of the 40-GEP as a more accurate predictor of metastatic risk. The study contributes to the growing body of literature on the use of gene expression profile testing in cutaneous cancers, emphasizing the need for further research in this area to improve patient care outcomes using 40-GEP testing.

## Introduction

Staging and categorization for cutaneous cancers act as guides to help in managing treatment options. As of now, there are several staging and categorization types used to predict the best course of action for curative procedural interventions. These methods include appropriate use criteria (AUC) for Mohs surgery [1], the American Joint Committee for Cancer eighth edition (AJCC8) staging manual [2], and the Brigham and Women’s Hospital (BWH) staging for cutaneous squamous cell carcinoma (SCC) [3]. The AUC is broken into zones and specific rules that guide when to use Mohs surgery for intervention. These zones include the H-zone (central face, eyelids, nose, lips, chin, ear, temple, genitalia hands, feet, nails, ankles, and nipples/areola [1]), M-zone (cheeks, forehead, scalp, neck, jawline, and pretibial surface [1]), and L-zone (trunk and extremities) [1]. SCC is appropriate to treat in zones H and M, whereas the L-zone requires size criteria, immunocompromised state, recurrence, aggressive tumor type, or other unique characteristics to be met [1]. Cutaneous skin cancers, such as SCCs, carry a known metastatic risk to patients, which can lead to morbidity and mortality [2-4]. Metastasis can be difficult to detect, but early metastasis detection can improve patient survival due to the early implication of suitable treatment [4]. The current staging systems are not fully adequate in predicting the metastatic risk profile in cutaneous SCC patients [5-6]. This results in misidentifying high-risk vs low-risk patients [5-6]. To better quantify the metastatic potential of SCCs, a 40-gene expression profile (40-GEP) was developed to better capture metastasis and metastatic risk as an independent predictor [4]. The 40-GEP classification includes prognostic groups based on metastatic risk: Class 1 (low risk), Class 2A (moderate risk), and Class 2B (high risk) [4]. Since these groups act as independent predictors of metastatic risk, they can be beneficial alone or in conjunction with staging techniques [4]. Our case report presents the successful usage of a 40-GEP as an independent predictor of metastatic risk, proving to be adjunctive and useful information, independent of AJCC8 and BWH in determining our patient’s metastatic risk.

## Case presentation

**Figure 1:**
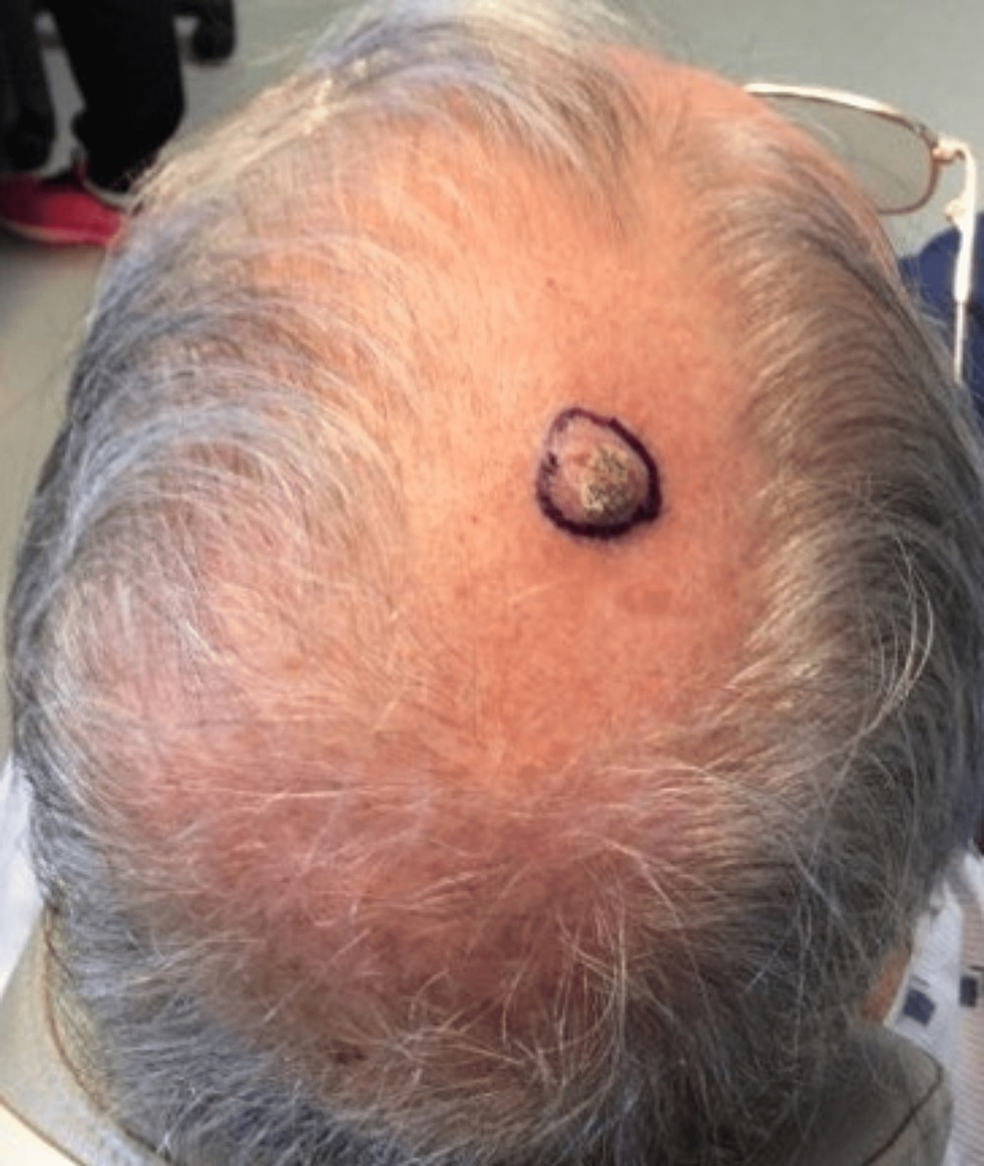
Cutaneous squamous cell carcinoma circled on the right superior parietal scalp

**Figure 2:**
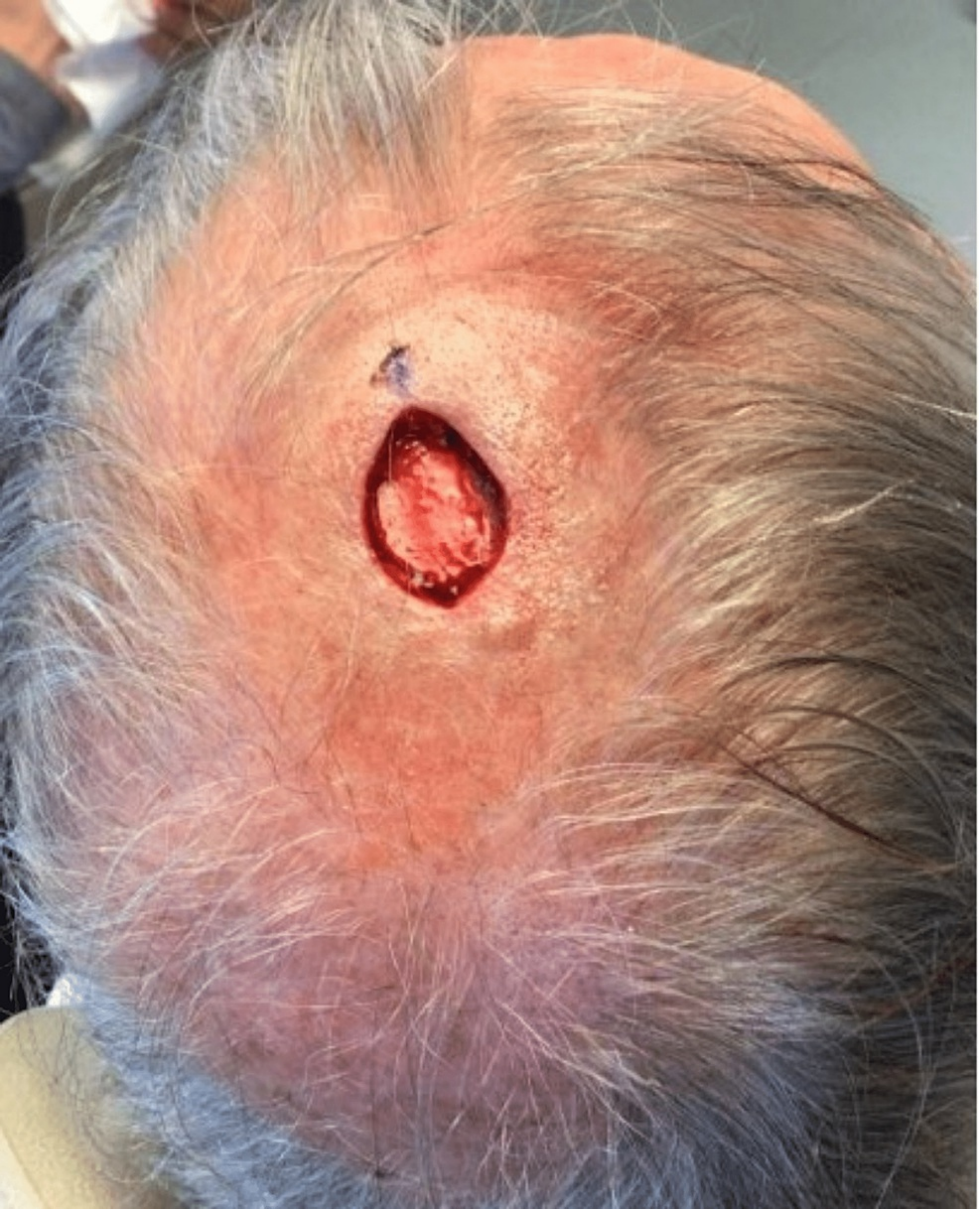
Mohs surgical defect after successful removal of cutaneous squamous cell carcinoma on the right superior parietal scalp

A 74-year-old male presented to the office for a Mohs procedure on a cutaneous SCC on the right superior parietal scalp (Figure 1). The patient had a history of melanoma and basal cell carcinoma, but no previous history of SCC. Initially, the lesion presented as an erythematous plaque with crust present for two months prior to biopsy. The pathology results showed a deeply infiltrative poorly differentiated SCC. No treatments were performed at this time, and the patient was referred for Mohs surgery, meeting the M-zone AUC. Based on the findings of the depth of invasion and poor differentiation, the AJCC8 staging was T3, and BWH staging was a T2b; however, his 40-GEP staging was a Class 1 (low risk) of metastasis within 3 years [4]. A Class 1 risk group has shown a rate of 91.6% metastatic-free survival at the three-year point [4]. The AJCC8 nodal metastatic risk was found to be approximately 13% for T2 and T3 groups, whereas the BWH T2b nodal metastatic risk was 24% [7]. The patient was successfully treated with Mohs surgery with no evidence of perineural invasion (Figure 2). This case highlights a discrepancy between current staging systems and risk classifications found in gene expression profile testing, which predicts the risk of metastasis independent of current staging systems. In this case, the patient was highly staged by standard staging systems, yet was considered low risk of metastasis with gene expression profile testing.

## Discussion

Our case highlights a discrepancy in current staging techniques and the results of 40-GEP testing. In this case, our patient had an AJCC8 staging was T3 (stage T2: tumor > 2 cm, but ≤ 4 cm in diameter, stage T3: tumor > 4 cm in diameter or minor bone erosion, perineural invasion or deep invasion) [2], and BWH staging was a T2B (staging that included two to three risk factors: > 2 cm lesion, poorly differentiated histology, and tumor invasion beyond the subcutaneous fat) [3]; however, his 40-GEP staging was Class 1 (low risk) < 7% risk of metastasis within three years [4-6]. This method uses a molecular algorithm of the gene expression profile of cutaneous SCCs with known outcomes [4-6].

The 40-GEP test stratifies primary cSCC patients having one or more clinicopathologic risk factors into three biological risk groups based on risk for regional, nodal, or distant metastasis (low = Class 1; moderate = Class 2A; high = Class 2B) [4]. Gene testing is an emerging field with the potential to better predict outcomes. We hope this case can add to the discussion and growing body of knowledge surrounding methods to better provide accurate patient metastatic risk outcomes through gene expression profile testing. In our case, the independent predictive quality of the 40-GEP was different than those risks based on current standard staging methods.

## Conclusions

In this case, a more accurate assessment of the state of disease was attained through 40-GEP testing. Traditional staging methods, including AJCC8 and BWH, overestimated our patient’s risk profile. More research needs to be completed surrounding gene testing and metastatic risk. Our goal with this case is to contribute to the growing body of literature surrounding the use of gene expression profile testing in cutaneous cancer.
